# Mycotoxin Profile and Phylogeny of Pathogenic *Alternaria* Species Isolated from Symptomatic Tomato Plants in Lebanon

**DOI:** 10.3390/toxins13080513

**Published:** 2021-07-22

**Authors:** Wassim Habib, Mario Masiello, Romy El Ghorayeb, Elvis Gerges, Antonia Susca, Giuseppe Meca, Juan M. Quiles, Antonio F. Logrieco, Antonio Moretti

**Affiliations:** 1 Laboratory of Mycology, Department of Plant Protection, Lebanese Agricultural Research Institute, P.O. Box 90-1965, Fanar 1202, Lebanon; whabib@lari.gov.lb (W.H.); egerges11@gmail.com (E.G.); 2 Department of Nutrition and Food Sciences, Faculty of Arts and Sciences, Holy Spirit University of Kaslik, P.O. Box 446, Jounieh 1200, Lebanon; romyghorayeb@gmail.com; 3 Institute of Science of Food Production—ISPA, Research National Council—CNR, Via Amendola, 122/O, 70126 Bari, Italy; antonella.susca@ispa.cnr.it (A.S.); antonio.logrieco@ispa.cnr.it (A.F.L.); Antonio.moretti@ispa.cnr.it (A.M.); 4 Department of Preventive Medicine, Nutrition and Food Science Area, University of Valencia, Avenida Vicent Andres Estelles s/n, 46100 Valencia, Spain; Giuseppe.Meca@uv.es (G.M.); Juan.Quiles@uv.es (J.M.Q.)

**Keywords:** multilocus gene sequencing, toxigenic fungi, *Alternaria alternata*, *Alternaria arborescens*, *Alternaria mali* morpho-species

## Abstract

The tomato is one of the most consumed agri-food products in Lebanon. Several fungal pathogens, including *Alternaria* species, can infect tomato plants during the whole growing cycle. *Alternaria* infections cause severe production and economic losses in field and during storage. In addition, *Alternaria* species represent a serious toxicological risk since they are able to produce a wide range of mycotoxins, associated with different toxic activities on human and animal health. Several *Alternaria* species were detected on tomatoes, among which the most important are *A. solani*, *A. alternata*, and *A. arborescens*. A set of 49 *Alternaria* strains isolated from leaves and stems of diseased tomato plants were characterised by using a polyphasic approach. All strains were included in the recently defined phylogenetic *Alternaria* section and grouped in three well-separated sub-clades, namely *A. alternata* (24 out of 49), *A. arborescens* (12 out of 49), and *A. mali* morpho-species (12 out of 49). One strain showed high genetic similarity with an *A.* *limoniasperae* reference strain. Chemical analyses showed that most of the *Alternaria* strains, cultured on rice, were able to produce alternariol (AOH), alternariol methyl ether (AME), altenuene (ALT) and tenuazonic acid (TA), with values up to 5634, 16,006, 5156, and 4507 mg kg^−1^, respectively. In addition, 66% of the strains were able to co-produce simultaneously the four mycotoxins investigated. The pathogenicity test carried out on 10 *Alternaria* strains, representative of phylogenetic sub-clades, revealed that they were all pathogenic on tomato fruits. No significant difference among strains was observed, although *A. alternata* and *A. arborescens* strains were slightly more aggressive than *A. mali* morpho-species strains. This paper reports new insights on mycotoxin profiles, genetic variability, and pathogenicity of *Alternaria* species on tomatoes.

## 1. Introduction

The tomato is one of the most important agricultural crops produced and consumed in Lebanon. Grown on about 3800 hectares, in open fields or in greenhouses, after potato the tomato is considered the second biggest agro-food crop in terms of production and consumption, with a volume of about 300,000 tons [1]. Almost all tomato production is consumed in Lebanon, and about 5000 tons per year are exported to the Arab gulf countries.

The tomato is highly susceptible to several fungal pathogens, especially necrotrophic agents such as *Alternaria* species [2,3], which are the causal agents of early blight, a destructive disease that compromises the yield [4].

*Alternaria* is a ubiquitous fungal genus that includes endophytic, saprophytic and pathogenic species commonly found in soil, air, food commodities, and on decaying plant tissues [5]. As plant pathogens, *Alternaria* species have been reported on important crops, including cereals, oil crops, ornamentals, vegetables and fruits [5,6]. *Alternaria* survives as mycelium or conidia on decaying plant debris for a long time, or as a latent infection in seeds [7]. Moreover, weakened or wounded tissues are more prone to being colonised by *Alternaria* species [7,8].

Several *Alternaria* species have been reported on tomato plants and fruits. In particular, *A. solani* is considered the most important pathogen associated with early blight disease of tomato and potato plants [9,10], whereas *A. arborescens* and *A. alternata* are considered to be the most important species associated with stem canker and black spots of tomato fruits [11,12,13]. Moreover, other *Alternaria* species have been reported to be isolated from symptomatic tomato plants or fruits, such as *A. brassicicola*, *A. tenuissima*, *A. consortialis*, *A. linariae*, and *A. grandis* [14,15,16]. Nevertheless, the pathogenic impact of *A. alternata* on tomatoes is controversially discussed, since some researchers considered both *A. solani* and *A. alternata* as causal agents of early blight disease [9], whereas others reported that *A. alternata* is only a secondary invader [10,17].

Based primarily on morphological characters, Simmons organised the genus complexity of *Alternaria* by launching a species–group concept, and identified more than 270 *Alternaria* morpho-species [18,19]. Recently, severe taxonomic studies, based on the analyses of informative genes, including the major allergen *alt-a1* (*alt-a1*), glyceraldehyde-3-phosphate dehydrogenase (*gpd*), translation elongation factor (*tef*), and calmodulin allowed the redefinition of *Alternaria* species into 27 phylogenetic sections [20,21]. All small-spored *Alternaria* species, such as *A. alternata, A. arborescens,* and *A. tenuissima*, were grouped together in the *Alternaria* section, in addition to several other species, all synonymised with *A. alternata* species, considered as type species [22]. Conversely, the large-spored *A. solani* was included in section *Porri* [22,23,24,25,26], while recently a new section, named *Omanenses*, has been proposed [27]. The importance of well-defined species boundaries in this genus is also related to the high capability of several *Alternaria* species to produce mycotoxins and define a species–specific risk profile [5,6,7,8,9,10,11,12,13,14,15,16,17,18,19,20,21,22,23,24,25,26,27,28].

Indeed, the occurrence of *Alternaria* species on tomatoes is often associated with the production of mycotoxins in the fruit, which can be dangerous to human health [29,30,31]. Several studies reported that the accumulation of these mycotoxins in agri-food products is influenced by several environmental conditions that affect both the pre-harvest mould development and the consequent mycotoxin production [32,33]. *Alternaria* species produce several toxic metabolites, such as alternariol (AOH), alternariol methyl ether (AME), altertoxin-I (AT-I), altertoxin-II (AT-II) and tenuazonic acid (TA) [5,13,29,34,35,36,37]. Wang et al. [34] reported AAL-toxin produced by *A. arborescens* [11] as phytotoxic compounds responsible for the apoptosis of tomato cells by inhibiting ceramide biosynthesis [38]. Studies have shown that TA was responsible for some changes in the oesophageal mucosa of mice [39], and was reported as a cytotoxic, phytotoxic, antitumoral, antiviral, antibiotic and antibacterial compound [40]. Furthermore, AT-I was identified as toxic in mice and mutagenic to mammalian cell lines [41], and AOH and AME were identified as genotoxic and mutagenic, with immune modulating effects [42]. In addition, more recently, Huybrechts et al. [43] associated *Alternaria* mycotoxins to colon rectal cancer in humans.

Despite the widespread occurrence of early blight on tomatoes in Lebanon, no data are currently available on the population of *Alternaria* species occurring on tomatoes. Therefore, in order to evaluate the *Alternaria* mycotoxin risk and achieve knowledge for improving the disease management strategy and increasing the quality of the production, this work aimed to: (a) identify the main *Alternaria* species causing early blight of tomatoes in Lebanon and establish their phylogenetic relationships by using a multilocus sequence approach; (b) define their mycotoxin profiles; (c) assess their pathogenicity on the tomato fruits.

## 2. Results

### 2.1. Phylogenetic Analysis

The sequences of each gene were aligned and cut at the ends to analyse a common fragment for all of the strains. In particular, for the *alt-a1* gene a fragment of about 440 nucleotides (nt) was considered; for the *gpd* gene a fragment of 510 nt, for the *calmodulin* gene a fragment of 750 nt, and for the *β-tubulin* gene a fragment of 500 nt. All genes were very informative for discriminating between *Alternaria* species, although *alt-a1* and *calmodulin* genes showed the highest degree of variability.

**Figure 1:**
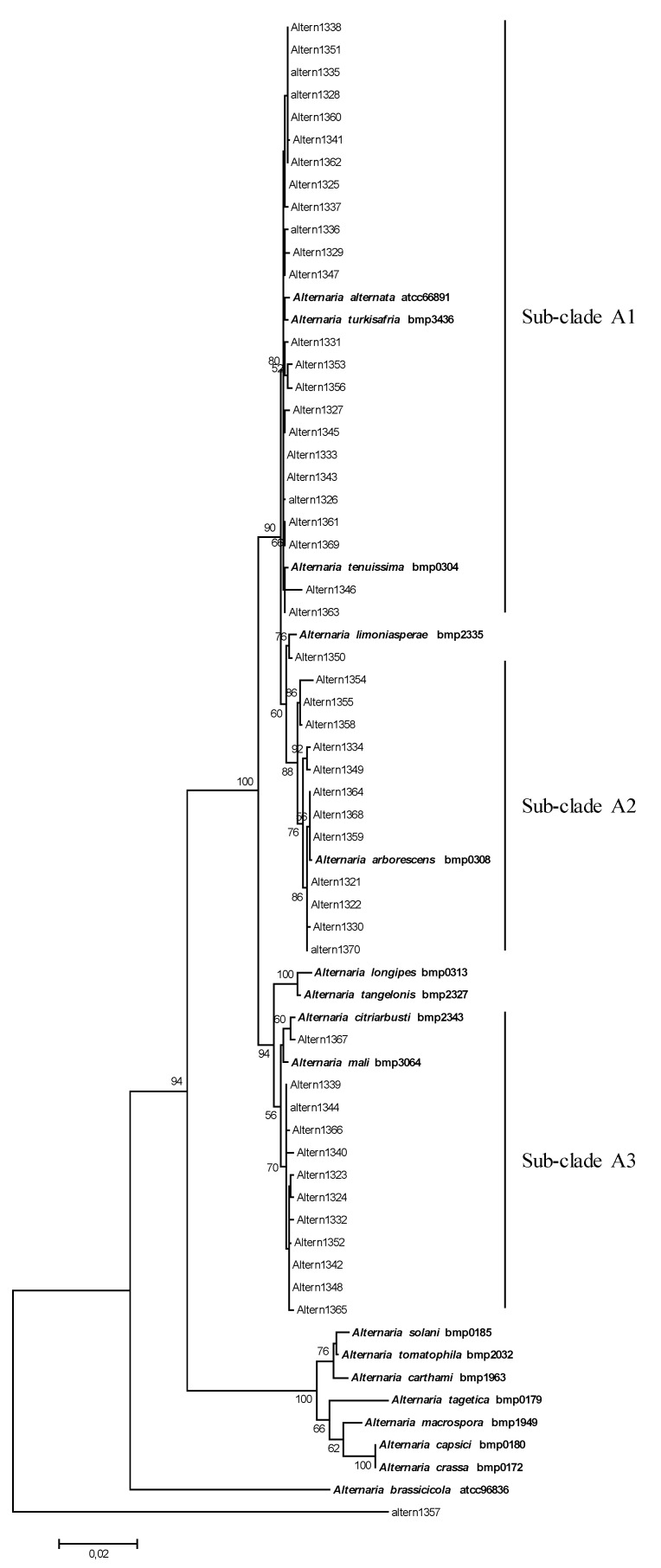
Phylogenetic tree generated and obtained by maximum likelihood method (bootstrap 1000 replicates) of combined *alt-a1*, *gpd*, *cal* and *tub* gene sequences of 50 fungal strains isolated from infected tomato plants.

The final sequence alignment of the combined dataset comprising 67 taxa included 49 *Alternaria* strains isolated from tomatoes, 17 *Alternaria* reference sequences and one strain of *Stemphylium* isolated also from tomatoes (Altern1357) as the outgroup taxon (Figure 1). The phylogenetic analysis of the concatenated sequences of the four fragments (*alt-a1*, *gpd*, *calmodulin* and *β-tubulin* genes) resulted in a phylogenetic tree that allowed us to discriminate between three well-separated clades, supported by high bootstrap values (>90), corresponding to Section *Alternaria*, Section *Porri* and Section *Brassicicola*.

All field strains grouped with the *Alternaria* reference sequences of the species belonged to the *Alternaria* section (Figure 1). In detail, 25 strains (sub-clade A1) grouped with *A. alternata*, *A. turkisafria*, and *A. tenuissima* species, showed a high homology with references and among themselves (Figure 1). The *Alternaria* strain Altern1350 clustered with the *A. limoniasperae* reference sequence (Figure 1). In the *Alternaria* section, a sub-clade (sub-clade A2) of 12 field strains clustered with the *A. arborescens* reference strain. In particular, three strains (Altern1364, Altern1368, and Altern1359) were very close to the reference (Figure 1). In this sub-group, a high variability was observed. In particular, the strains Altern1354, Altern1355 and Altern1358, were similar among them, and further away from *A. arborescens* reference strains, due to an insertion of 8 nt in the *Calmodulin* fragment. In the sub-clade A3, including *A. longipes*, *A. tangelonis*, *A. citriarbusti*, *A. mali* reference strains, one strain (Altern1367) was very close to *A. citriarbusti*; 11 strains, showing high homology among them, were closely related to both *A. mali* and *A. citriarbusti* references but did not cluster with any reference strain.

### 2.2. Mycotoxin Production

Thirty-five *Alternaria* strains were selected as representative of the three phylogenetic sub-clades (Figure 1) to evaluate their capacity to produce AME, AOH, ALT, and TA. The mycotoxin production of each strain and the mean values of each *Alternaria* phylogenetic sub-clade are reported in Table 1. The majority of the strains tested produced AOH (94.3%), AME (94.3%), and ALT (91.4%), whereas TA was produced by 68.6% of the strains tested. In addition, as a reason of particular concern, 65.7% of the strains were able to co-produce simultaneously the four mycotoxins investigated.

In the *A. alternata* group (sub-clade A1), with the exception of the *Alternaria* strain Altern1360, that produced only TA (2035 mg kg^−1^), all strains were able to produce simultaneously AOH, AME, and ALT with mean values of 1422, 4576, and 1498 mg kg^−1^, respectively. However, the mycotoxin quantities produced were dramatically variable for all mycotoxins, ranging between 26 and 4567, 20 and 15,070, and 1 and 5156 mg kg^−1^, for AOH, AME, and ALT, respectively (Table 1). These data showed a high intraspecific variability within this group. Tenuazonic acid was produced by 10 out of 16 strains included in this group, with values very variable, ranging between 145 and 4442 mg kg^−1^, with a mean value of 1890 mg kg^−1^. The strain Altern1350, showing a high phylogenetic homology with the *A. limoniasperae* bmp2335 strain, produced a low quantity of AME (102 mg kg^−1^) and ALT (4 mg kg^−1^).

All of the strains belonging to sub-clade A2, assigned to *A. arborescens* species-group, with the exception of Altern1354 that produced only AOH (3501 mg kg^−1^), were able to produce the mycotoxins analysed. As observed in sub-clade A1, the quantity of mycotoxins was very variable, ranging between 27 and 5634 mg kg^−1^ (mean value 2075) for AOH, between 25 and 16,006 mg kg^−1^ (mean value 4761) for AME, between 28 and 1560 mg kg^−1^ (mean value 529) for ALT, and between 75 and 3777 mg kg^−1^ (mean value 1290) for TA.

With regard to *Alternaria* strains included in the sub-clade A3, all strains were able to produce AOH and AME, with values ranging between 92 and 2367 mg kg^-1^ (mean value 802) and between 23 and 10,998 mg kg^−1^ (mean value 5967), respectively. For ALT and TA production, only one out of six strains (Altern1344) and two out of six (Altern1342, Altern1344), were not producers.

Among the four mycotoxins, AME was the highest mycotoxin produced in all phylogenetic groups, with values up to 16,006 mg kg^−1^ (*A. arborescens* Altern1349 strain) and ranging from 20 to 16,006 mg kg^−1^. Tenuazonic acid was the least mycotoxin produced, since 37% of *A. alternata* strains, 16% of *A. arborescens* strains, and 33% of *A. mali* morpho-species strains did not produce it (Table 1).

### 2.3. Pathogenicity Test

**Figure 2:**
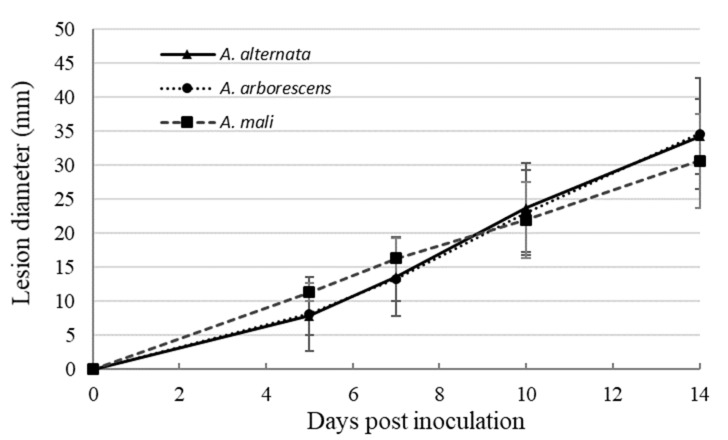
Mean values of the progression of the lesions caused on tomato fruits by a set of 10 *Alternaria* strains included in *A. alternata* (4 strains), *A. arborescens* (3 strains), and *A. mali* morpho-species (3 strains) sub-clades. Bars represent the standard errors.

The pathogenicity test on Grappe Randala tomato cultivar revealed that *Alternaria* representative strains of each sub-clade are all pathogenic on tomato fruits (Figure 2). During the first week of evaluation, the lesions caused by the *Alternaria* strains phylogenetically grouped in *A. mali* sub-clade (sub-clade A3) were slightly larger than lesions caused by *Alternaria* strains grouped in *A. alternata* and *A. arborescens* sub-clades (Figure 2). However, after 14 days, *A. alternata* and *A. arborescens* species produced larger lesions (34.3 mm and 34.7 mm, respectively) than the *A. mali* morpho-species (30.7 mm). Nevertheless, no significant difference between the strains of each sub-clade was observed.

## 3. Discussion

*Alternaria* is an economically important fungal genus able to colonise a wide range of crops, including cereals, fruits and vegetables [44]. Severe *Alternaria* infections cause both economic losses for producers and health risk for consumers, due to the accumulation of toxic metabolites in agri-food products [45]. Several *Alternaria* species, such as *A. solani*, *A. alternata*, *A. arborescens*, and *A. tenuissima* have been reported worldwide as causal agents of important tomato diseases, as early blight or leaf spot diseases [8,46]. Tomato plants can be colonised by *Alternaria* species during the whole growth cycle, with damage to leaves, stems and fruits. In particular, at fruit growth time, environmental conditions suitable for *Alternaria* infections can cause severe yield losses [47,48,49].

For a long time, the most important concern of phytopathogenic *Alternaria* species was the yield and economic losses in pre- and post-harvest conditions. In the last years, more emphasis has been posed on the mycotoxigenic risk of *Alternaria* species, since several studies have shown the haemato-toxic, genotoxic, and mutagenic activities of the most important *Alternaria* mycotoxins: AOH, AME, and TA [41,42,45]. In addition, the investigations carried out worldwide on the mycotoxin profile at a species level have provided information widely used as a chemotaxonomic approach along the genus. Indeed, chemotaxonomy has been considered a highly useful tool to be integrated in a poly-phasic approach for defining species boundaries in *Alternaria* [21,28,50,51]. Therefore, in the present study, a set of representative *Alternaria* strains, isolated from tomato plants grown in greenhouses in Lebanon, were characterized by using a polyphasic approach aimed at evaluating the genetic, chemical and pathogenic differences among *Alternaria* species detected on tomatoes. The phylogenetic relationship among *Alternaria* strains was evaluated based on the sequence analyses of four informative genes, *gpd*, *alt-a1*, *calmodulin*, and *β-tubulin* [21,24]. All *Alternaria* strains were genetically grouped in the recently defined *Alternaria* section [22], in which 35 small-spore morphological *Alternaria* species were all synonymised with *A. alternata* species based on phylogenetic studies [22].

*Alternaria alternata* was the most frequently detected species, with a frequency of around 50%. Similar results were previously reported in studies carried out in other countries, such as Egypt [52], Algeria [15], and Russia [16]. However, in Russian regions, *A. alternata* was detected alone or in a complex with *A. infectoria* and *A. solani*, which represented 30% of the strains detected [16]. In this study, 25% of the field strains were identified as *A. arborescens*. Since these strains were isolated from leaves and stems showing *Alternaria* symptoms, the importance of the role of *A. arborescens* species as a cause of stem canker in tomatoes in Lebanon has been confirmed.

A group of 12 *Alternaria* strains (25%), genetically very close to *A. citriarbusti* and *A.mali* reference strains, were for the first time isolated from infected tomato tissues. In the last years, genetic investigations have allowed the detection of *A. mali* morpho-species on different crops, including wheat [53,54], and *Cakile maritima*, a spontaneous halophyte plant growing on Tunisian sandy coasts [55]. Surprisingly, no *A. solani* strain was detected in Lebanon, although this *Alternaria* species has been reported worldwide as the most predominant pathogen associated with solanaceous plants, such as potatoes and tomatoes [9,10].

We investigated the capability of *Alternaria* strains isolated from leaves and stems of tomato plants to colonise fruits. Indeed, in addition to the phytopathogenic role of *Alternaria* species, nowadays the most important concern is the toxicological risk of *Alternaria* mycotoxins and their accumulation on fruits and vegetable-based products. All tested *Alternaria* representative strains, belonging to the three phylogenetic sub-clades, showed a great capability to colonise fruits. Pathogenicity tests carried out in this study showed that the level of aggressiveness of all *Alternaria* strains showed a similar degree of pathogenicity, with no significant differences between the species. However, *A. alternata* and *A. arborescens* species seemed more capable of infecting tomato fruits, suggesting that these two species are more prone to colonising them. Wenderoth et al. [56] showed a strict correlation between AOH production and the capability of *A. alternata* strains to colonise tomatoes, apples and citrus fruits, supporting the role of AOH as a virulence factor [57,58]. The results of our pathogenicity assay can corroborate this role, since all strains tested, all producing AOH in vitro conditions, were able to colonize tomato tissues.

The potential capacity of *Alternaria* strains to produce mycotoxins has also been evaluated. Almost all strains were able to synthesise the four mycotoxins tested (TA, AME, ALT and AOH), although a great quantitative variability in their production was observed. This is due to both inter- and intra-species differences, as well as to the peculiar conditions required by each species for the optimal production. A great variability to produce mycotoxin has been observed, although almost all strains were able to produce mycotoxins. Alternariol monomethyl ether was the most dominantly produced toxin (up to 16006 mg kg^−1^), since all *Alternaria* tested strains, except a single *A. alternata* strain and a single *A. arborescens* strain, were able to synthetise this mycotoxin. The toxic effects of this mycotoxin could be emphasised by the occurrence of sulphate and glucoside conjugate forms that *Alternaria* strains were showed to produce when cultured on tomatoes [59]. Tenuazonic acid, reported as the most produced mycotoxin by *Alternaria* species [52,60] was produced by around 70% of the *Alternaria* strains, with a dramatic variability of production levels (up to 4507 mg kg^−1^). In addition, the co-production of TA, AME, ALT and AOH by most of the strains should be taken into account, since the toxicological risk caused by possible additive and/or synergistic effects on target organisms could be exacerbated [61]. These values are higher than those found naturally in tomatoes (mg/kg vs. ug/kg) due to both ideal conditions of temperature and humidity used, and inoculated substrate that trigger the optimal synthesis of the mycotoxins targeted. Indeed, when grown in vivo on autoclaved rice, in which no mechanical barriers occur and many of the grain defences, that are thermolabile, have been suppressed, the strains could synthesize mycotoxins, providing a sort of ontological risk evaluation.

The identification of a set of *Alternaria* strains, using a polyphasic approach, can provide useful knowledge on the *Alternaria* population affecting tomato plants in the most important Lebanese areas allocated to solanaceous crops. *Alternaria alternata* and *A. arborescens* were the most dominant species detected, both showing a strict association with the destructive tomato disease in Lebanon. In addition, the great capability of these strains to produce mycotoxins poses evidence of the potential toxicological risk of tomato-based products, largely consumed worldwide. Finally, these results will be functional in developing integrated strategies for disease management and breeding programs to reduce *Alternaria* infection on tomatoes and highlight that the mycotoxin profile of *Alternaria* species associated with tomato disease can greatly vary according with geographical areas, likely due to the continuous changes of environmental conditions.

## 4. Materials and Methods

### 4.1. Tomato Sampling and Fungal Isolation

Samples of leaves and stems showing characteristic symptoms of early blight were collected during the 2017 crop season from tomato plants, grown under greenhouses, in the four most important Lebanese tomato producing areas: Metn (6 samples), Jbeil (48 samples), North District (26 samples), and Chouf (11 samples). After a surface-disinfection with 2% sodium hypochlorite solution for 2 min, 70% ethanol for 30 s, and two washings with sterile distilled water for 1 min, portions of tomato plant shoots were dried on sterile filter paper in a laminar flow cabinet. Small pieces (2 × 2 mm) were then placed on potato dextrose agar (PDA, Laboratorios Conda S.A., Madrid, Spain) amended with 0.10 g L^−1^ streptomycin sulphate salt and 0.05 g L^−1^ neomycin. Petri dishes were incubated for 5 days at 25 ± 1 °C under an alternating light/darkness cycle of a 12 h photoperiod. *Alternaria* colonies originating from tomato tissues were selected to obtain mono-conidial cultures [62]. A set of 49 representative *Alternaria* strains (Metn: 3 strains; Jbeil: 24 strains; North District: 15 strains; Chouf: 7 strains), together with one outgroup *Stemphylium* strain (ALT-1357) were selected for phylogenetic and mycotoxin profile analyses.

### 4.2. DNA Extraction

Genomic DNA was extracted and purified, according to Murray and Thompson [63], from two-day-old colonies grown at 25 ± 1 °C on cellophane disks overlaid on PDA. Briefly, mycelium was collected by scraping the cellophane, powdered under liquid nitrogen and added with 600 μL of CTAB buffer (100 mM Tris-Cl, pH 8.0; 1.4 M NaCl; 20 mM EDTA, pH 8.0; 2% cetyldimethylethylammonium bromide (*w/v*); 0.2% β-mercaptoethanol (*v/v*)). The samples were frozen and de-frozen three times using liquid nitrogen and a water bath at 75 °C, and then incubated at 75 °C for 1 h. Following the use of 1 volume chloroform, the clear supernatant was transferred to a new tube and precipitated with 2 volumes of isopropanol at –80 °C per 30 min. The samples were then centrifuged at 14,000 rpm for 15 min, and the nucleic acid pellet was washed with cold 70% ethanol stored at −20 °C, air-dried, and dissolved in TE (10 mM Tris-Cl; 1 mM EDTA, pH 8). The suspension was then added with 0.1 μg μL^−1^ DNase-free pancreatic RNase (Sigma-Aldrich, Milan, Italy) and incubated for 2 h at 37 °C, precipitated by the addition of 0.6 volume of 5 M ammonium acetate and 2 volumes of cold absolute ethanol. The final DNA pellet, washed with 70% ethanol and air-dried, was dissolved in nuclease free water and stored at –80 °C until use. Quantity and integrity of DNA were checked with Thermo-Scientific Nanodrop (LabX, Midland, ON, Canada), and on 0.8% agarose gel, by electrophoretic separation, using a standard 1 kb DNA Ladder (Invitrogen, Thermo Fisher Scientific, Carlsbad, CA, USA).

### 4.3. PCR Amplification

For each *Alternaria* strain, four informative target genes, *allergen alt 1a* (*alt-a1*), *glyceraldeyde-3-phosphate dehydrogenase* (*gpd*), *calmodulin* (*calm*), and *β-tubulin* (*tub*), were amplified with the primer pairs alt-for/alt-rev [64], gpd1/gpd2 [65], CALDF1/CALDR1 [24], and T1/T2 [66,67], respectively.

Amplification of *alt-a1* gene was performed using a Takara (Takara Bio Inc., Otsu, Shiga, Japan) kit in mixture with 1× Takara PCR Buffer, 0.075 μL of Hot Master Taq DNA Polymerase (1 U/μL), 0.3 μL of dNTPs (10 mM), 0.45 µM of each primer and 15 ng of DNA template. Amplification of *gpd* genes was performed using GoTaq G2 Colorless Master Mix (Promega Corporation, Madison, WI, USA) in mixture with a final concentration of 1× ready Master Mix, 2 mM MgCl_2,_ 0.4 µM of each primer, and 25 ng of DNA template. *Calmodulin* and *tub* genes were amplified using 50 ng of DNA template, 0.45 µL of each primer (10 mM), 0.3 µL of dNTPs (10 mM), and 0.075 µL of Hot Master Taq DNA Polymerase (1 U/µL; 5 Prime).

Amplifications were carried out in the GeneAmp PCR System 9700 thermal cycler (Applied Biosystems, Foster City, CA, USA). The PCR reactions for *alt-a1*, *gpd*, and *tub* were carried out by using thermal cycler parameters reported by Somma et al. [21]. To amplify the *calm* gene, thermal cycler parameters were: initial denaturation for 4 min at 95°C followed by 35 cycles of 95 °C for 30 s, 58.5 °C for 30 s, 72 °C for 60 s, and a final extension for 5 min at 72 °C. For each reaction, a no-template control was included to ascertain the absence of contamination. The PCR products, stained with GelRed^®^ (GelRed^®^ Nucleic Acid Gel Stain, 10,000X, Biotium Inc., Fremont, CA, USA) were visualised with UV after electrophoretic separation in 1× TAE buffer, on 1.5% agarose gel and sized by comparison with 100 bp DNA Ladder (Invitrogen, Thermo Fisher Scientific, Carlsbad, CA, US).

### 4.4. Sequencing and Phylogenetic Analysis

Each PCR product was purified with the enzymatic mixture Exo/FastAP (Exonuclease I, FastAP thermosensitive alkaline phosphatase, Thermo Fisher Scientific, Vilnius, Lithuania) and then sequenced with a Big Dye Terminator Cycle Sequencing Ready Reaction Kit (Applied Biosystems, Foster City, CA, USA), according to the manufacturer’s recommendations for both strands of each gene. The fragments were purified by filtration through Sephadex G-50 (5%) (Sigma-Aldrich, Saint Louis, MO, USA) and sequenced in an “ABI PRISM 3730 Genetic Analyzer” (Applied Biosystems, Foster City, CA, USA). Partial sequences were assembled using the BioNumerics v. 5.1 software (Applied Maths, Inc., Austin, TX, USA). A phylogenetic tree of concatenated gene sequences was generated by using maximum a likelihood statistical method and bootstrap analysis (1000 replicates, removing gaps) with MEGA7 [68].

Gene sequences of the reference strains *A. alternata* E.G.S.34.016, *A. tenuissima* E.G.S.34.015, *A. arborescens* E.G.S.39.128, *A. brassicicola* ATCC96836, *A. capsici* BMP0180, *A. carthami* BMP1963, *A. citriarbusti* BMP2343, *A. crassa* BMP0172, *A. limoniasperae* BMP2335, *A. longipes* BMP0313, *A. macrospora* BMP1949, *A. mali* BMP3064, *A. solani* BMP0185, *A. tagetica* BMP0179, *A. tangelonis* BMP2327, *A. tomotophila* BMP2032, and *A. turkisafria* BMP3436 were downloaded from the National Center for Biotechnology Information (NCBI) and “Alternaria Genomes Database” (AGD) and included in the phylogenetic analysis.

Sequences derived in this study were deposited in the GenBank database (Supplemental Table S1).

### 4.5. Mycotoxin Production and Analysis

Thirty-five *Alternaria* strains selected as representative of the population were inoculated, using three small plugs from 1-week-old colonies, on 30 g of autoclaved rice with 40% moisture in 250 mL of flasks [69]. Flasks were incubated for 21 days at 25 °C in darkness, and then the samples were finely ground with an Oster Classic grinder (220–240 V, 50/60 Hz, 600 W; Madrid, Spain).

The method used for mycotoxin extraction was based on that described by Rubert et al. [70], with some modifications. Briefly, 5 g of each ground sample was transferred into a 50 mL plastic tube containing 25 mL of methanol. The extraction was carried out using an Ultra Ika T18 basic Ultra-turrax, Ika, (Staufen, Germany), for 3 min at 1000 rpm. The extract was centrifuged at 4000× rpm for 5 min at 5 °C. One millilitre of the supernatant was filtered through a 0.22 µm nylon filter and diluted before injection into high performance liquid chromatography associated with a diode array detector (LC-DAD). Tenuazonic acid, AME and AOH standards were provided by SIGMA Chemical Company (St. Louis, MO, USA). Altenuene standard was provided by ChemCruz (Dallas, TX, USA). The method used for mycotoxin analysis was based on that described by Myresiotis et al. [71], with some modifications. Tenuazonic acid, AME, ALT and TA were determined using Merk HPLC with a LC-DAD L-7455 (Merk, Darmstadt, Germany) at 256 nm and a Hitachi Software Model D-7000 version 4.0 ((Merck KGaA, Darmstadt, Germania) was used for data analysis. A Gemini C18 column (Phenomenex, Torrance, CA, USA) 4.6 × 150 mm, 3 µm particle size was used as the stationary phase. The mobile phase consisted of two eluents, namely eluent A (water with 50 µL/L trifluoroacetic acid) and eluent B (acetonitrile with 50 µL/L trifluoroacetic acid). A gradient program with a constant flow rate of 1 mL/min was used, starting with 90% A and 10% B, reaching 50% B after 15 min and 100% B after 20 min. Then, 100% B was maintained for 1 min. Thereafter, the gradient was returned to 10% B in 1 min and allowed to equilibrate for 3 min before the next analysis [71].

This analytical method was validated by calculating, for each mycotoxin analysed, linearity, recovery, repeatability, reproducibility, limits of detection (LOD), limits of quantification (LOQ), and matrix effect. Linearity was evaluated using paired matrix calibrations in triplicate at concentrations between 5 and 500 μg kg^−1^. All of the mycotoxins showed good linearity in the working range, with resolution determination coefficients (*R*^2^) greater than 0.9922. LODs and LOQs were calculated by analysing blank samples enriched with the standard mycotoxins. These two parameters were assessed as the lowest concentration of the evaluated mycotoxins that gave a chromatographic peak at a signal-to-noise ratio (S/N) of 3 and 10 for LOD and LOQ, respectively. The recovery value was carried out in triplicate for three consecutive days using three addition levels: LOQ, 2 × LOQ, and 10 × LOQ. To calculate the matrix effect (ME), the calibration slope from the matrix calibration curve was divided by the slope of the standard calibration curve and multiplied by 100. All of these results are shown in Table 2.

In particular, LOD and LOQ of the method used were of 0.01 and 0.1 mg kg^−1^, respectively.

### 4.6. Pathogenicity Test

The pathogenicity test for a set of 10 previously identified isolates, selected as representative of the phylogenetic groups, was carried out on six-week-old plants of ‘Grappe Randala’ tomato cultivar grown in the experimental greenhouse of the agricultural research station of the Holy Spirit University of Kaslik, located in Hboub, Jbeil. In detail, a conidial suspension was prepared for each isolate by gently scrapping the surface of a 10-day-old culture grown on PDA using a bacteriological loop after adding sterile distilled water containing Tween 20 (0.05%), and then filtered on glass fibre and diluted to a final concentration of 10^6^ spores mL^−1^. For each isolate, 10 fruits growing on two plants were inoculated by placing 2 µL of the suspension on an injury artificially made by the tip of a sterile blade. A negative control was conducted by inoculating 10 fruits with sterile distilled water containing Tween 20 (0.05%). The pathogenicity of the isolates was determined by measuring the diameter of the observed lesions each three to four days for a period of two weeks.

## Figures and Tables

**Table 1:** Production of alternariol (AOH), alternariol methyl ether (AME), altenuene (ALT), and tenuazonic acid (TA), expressed in (mg kg^−1^), evaluated on 35 strains of *Alternaria* species from tomato leaves and stems affected by early blight.

Strain	*Alternaria* Species	Mycotoxin (mg·kg^−1^)			
		AOH	AME	ALT	TA
Altern1325	*A. alternata*	26	20	1	4442
Altern1327 *	*A. alternata*	908	6900	1538	2658
Altern1328	*A. alternata*	4567	4967	1025	Nd **
Altern1329 *	*A. alternata*	1271	4967	558	Nd
Altern1331	*A. alternata*	102	516	462	603
Altern1333	*A. alternata*	84	82	1959	1331
Altern1336	*A. alternata*	126	855	74	532
Altern1337	*A. alternata*	676	632	4097	Nd
Altern1341 *	*A. alternata*	4002	9172	214	3938
Altern1343 *	*A. alternata*	131	660	2687	Nd
Altern1345	*A. alternata*	1646	11,021	394	Nd
Altern1351	*A. alternata*	3090	8678	1114	145
Altern1353	*A. alternata*	441	2718	1238	2066
Altern1360	*A. alternata*	Nd	Nd	Nd	2035
Altern1362	*A. alternata*	3062	15,070	5156	1155
Altern1346	*A. alternata*	1199	2385	1950	Nd
Mean value		1422	4576	1498	1890
Min		26	20	1	145
Max		4567	15,070	5156	4442
Altern1350	*A. limoniasperae*	Nd	102.51	3.86	Nd
Altern1321	*A. arborescens*	27	25	42	2083
Altern1322	*A. arborescens*	333	249	134	283
Altern1330 *	*A. arborescens*	2059	6395	570	Nd
Altern1334	*A. arborescens*	610	405	387	2713
Altern1349	*A. arborescens*	5634	16,006	735	1251
Altern1354	*A. arborescens*	3501	Nd	Nd	Nd
Altern1355 *	*A. arborescens*	4612	5737	597	1008
Altern1358 *	*A. arborescens*	264	142	28	456
Altern1359	*A. arborescens*	1786	3026	550	754
Altern1364	*A. arborescens*	3271	6662	1560	496
Altern1368	*A. arborescens*	69	860	328	75
Altern1370	*A. arborescens*	2731	12,868	894	3777
Mean value		2075	4761	529	1290
Min		27	25	28	75
Max		5634	16,006	1560	3777
Altern1323 *	*A. mali*	92	23	286	4507
Altern1332	*A. mali*	254	10,182	1153	649
Altern1340 *	*A. mali*	384	893	2046	1896
Altern1342	*A. mali*	2367	10,998	2722	Nd
Altern1344	*A. mali*	796	6593	Nd	Nd
Altern1352 *	*A. mali*	915	7111	1326	452
Mean value		802	5967	1507	1876
Min		92	23	286	452
Max		2367	10,998	2722	4507

*^:^ *Alternaria* strains used in pathogenicity test; **: Nd: Not detected.

**Table 2:** Analytical parameters calculated for each mycotoxin tested.

Mycotoxin	LOD (mg/kg)	LOQ (mg/kg)	Recovery (%)	ME (%)
**AOH**	0.01	0.1	71	79
**AME**	0.01	0.1	70	78
**ALT**	0.01	0.1	62	75
**TA**	0.01	0.1	65	73

## Data Availability

The data presented in this study are available in “Habib, W.; Masiello, M.; El Ghorayeb, R.; Gerges, E.; Susca, A.; Meca, G.; Quiles, J.M.; Logrieco, A.F.; Moretti, A. Mycotoxin Profile and Phylogeny of Pathogenic Alternaria Species Isolated from Symptomatic Tomato Plants in Lebanon. Toxins 2021, 13, x. https://doi.org/10.3390/xxxxx”.

## Supplementary Materials

The following are available online at https://www.mdpi.com/article/10.3390/toxins13080513/s1, Table S1: GenBank accession numbers of *Alternaria* strain sequences obtained in the present study.
