# Supplementary material for: Mycotoxin Profile and Phylogeny of Pathogenic Alternaria Species Isolated from Symptomatic Tomato Plants in Lebanon
Source: Toxins (Basel). 2021 Jul 22;13(8):513. doi: 10.3390/toxins13080513 (PMC8402471; doi:10.3390/toxins13080513)
Supplement: Supplementary file 1 [file toxins-13-00513-s001.zip › toxins-1303264-supplementary.pdf]

Table S1. GenBank accession numbers of *Alternaria* strain sequences obtained in the present study.

Strain	Species	Accession Number			
		Alt-a1	*tub*	*calm*	*gpd*
Altern1321	*Alternaria arborescens*	OU407647	OU407696	OU407745	OU407794
Altern1322	*Alternaria arborescens*	OU407648	OU407697	OU407746	OU407795
Altern1323	*Alternaria mali*	OU407649	OU407698	OU407747	OU407796
Altern1324	*Alternaria mali*	OU407650	OU407699	OU407748	OU407797
Altern1325	*Alternaria alternata*	OU407651	OU407700	OU407749	OU407798
Altern1326	*Alternaria alternata*	OU407652	OU407701	OU407750	OU407799
Altern1327	*Alternaria alternata*	OU407653	OU407702	OU407751	OU407800
Altern1328	*Alternaria alternata*	OU407654	OU407703	OU407752	OU407801
Altern1329	*Alternaria alternata*	OU407655	OU407704	OU407753	OU407802
Altern1330	*Alternaria arborescens*	OU407656	OU407705	OU407754	OU407803
Altern1331	*Alternaria alternata*	OU407657	OU407706	OU407755	OU407804
Altern1332	*Alternaria mali*	OU407658	OU407707	OU407756	OU407805
Altern1333	*Alternaria alternata*	OU407659	OU407708	OU407757	OU407806
Altern1334	*Alternaria arborescens*	OU407660	OU407709	OU407758	OU407807
Altern1335	*Alternaria alternata*	OU407661	OU407710	OU407759	OU407808
Altern1336	*Alternaria alternata*	OU407662	OU407711	OU407760	OU407809
Altern1337	*Alternaria alternata*	OU407663	OU407712	OU407761	OU407810
Altern1338	*Alternaria alternata*	OU407664	OU407713	OU407762	OU407811
Altern1339	*Alternaria mali*	OU407665	OU407714	OU407763	OU407812
Altern1340	*Alternaria mali*	OU407666	OU407715	OU407764	OU407813
Altern1341	*Alternaria alternata*	OU407667	OU407716	OU407765	OU407814
Altern1342	*Alternaria mali*	OU407668	OU407717	OU407766	OU407815
Altern1343	*Alternaria alternata*	OU407669	OU407718	OU407767	OU407816
Altern1344	*Alternaria mali*	OU407670	OU407719	OU407768	OU407817
Altern1345	*Alternaria alternata*	OU407671	OU407720	OU407769	OU407818
Altern1346	*Alternaria alternata*	OU407672	OU407721	OU407770	OU407819
Altern1347	*Alternaria alternata*	OU407673	OU407722	OU407771	OU407820
Altern1348	*Alternaria mali*	OU407674	OU407723	OU407772	OU407821
Altern1349	*Alternaria arborescens*	OU407675	OU407724	OU407773	OU407822
Altern1350	*Alternaria limoniasperae*	OU407676	OU407725	OU407774	OU407823
Altern1351	*Alternaria alternata*	OU407677	OU407726	OU407775	OU407824
Altern1352	*Alternaria mali*	OU407678	OU407727	OU407776	OU407825
Altern1353	*Alternaria alternata*	OU407679	OU407728	OU407777	OU407826
Altern1354	*Alternaria arborescens*	OU407680	OU407729	OU407778	OU407827
Altern1355	*Alternaria arborescens*	OU407681	OU407730	OU407779	OU407828
Altern1356	*Alternaria alternata*	OU407682	OU407731	OU407780	OU407829
Altern1358	*Alternaria arborescens*	OU407683	OU407732	OU407781	OU407830
Altern1359	*Alternaria arborescens*	OU407684	OU407733	OU407782	OU407831
Altern1360	*Alternaria alternata*	OU407685	OU407734	OU407783	OU407832
Altern1361	*Alternaria alternata*	OU407686	OU407735	OU407784	OU407833
Altern1362	*Alternaria alternata*	OU407687	OU407736	OU407785	OU407834
Altern1363	*Alternaria alternata*	OU407688	OU407737	OU407786	OU407835
Altern1364	*Alternaria arborescens*	OU407689	OU407738	OU407787	OU407836
Altern1365	*Alternaria mali*	OU407690	OU407739	OU407788	OU407837
Altern1366	*Alternaria mali*	OU407691	OU407740	OU407789	OU407838
Altern1367	*Alternaria citriarbusti*	OU407692	OU407741	OU407790	OU407839
Altern1368	*Alternaria arborescens*	OU407693	OU407742	OU407791	OU407840
Altern1369	*Alternaria alternata*	OU407694	OU407743	OU407792	OU407841
Altern1370	*Alternaria arborescens*	OU407695	OU407744	OU407793	OU407842
